# 1257. The Impact of Communication Type on Acceptance Rates of Antimicrobial Stewardship Interventions

**DOI:** 10.1093/ofid/ofad500.1097

**Published:** 2023-11-27

**Authors:** Faiza Morado, Neha Nanda

**Affiliations:** Keck Medical Center of USC, Pasadena, California; USC, los angeles, California

## Abstract

**Background:**

While face-to-face communication (ie. “handshake stewardship” (HS)) has been shown to improve antimicrobial stewardship program (ASP) intervention acceptance rates, this strategy is resource intensive. Therefore, systems unable to conduct HS must identify the optimal mode of communication for ASP recommendations. This study evaluated the effects of communication type on ASP intervention acceptance.

**Methods:**

An infectious disease physician and ASP pharmacist met Monday through Friday to assess appropriateness of select antimicrobials. ASP recommendations were delivered through direct communication via telephone conversations or passive communication via text-page. A text page consisted of a message limited to 240 characters sent directly to the primary team. We performed a retrospective review of all interventions and associated acceptance rates, antimicrobials and antimicrobial indications between January 1^st^, 2023 to March 31^st^, 2023. The primary outcome evaluated was ASP intervention acceptance rate.

**Results:**

Recommendations were delivered through telephone conversations in 267/454 (58.8%) cases compared to 187/454 (41.2%) cases in which recommendations were provided via text-page. We found that direct communication delivered telephonically resulted in a significant increase in ASP intervention acceptance rate compared to passive communication via text page (82.8% vs 73.8%, respectively; p=0.02). This resulted in 884 and 552 days of antimicrobial therapy saved in the telephonic and text-page group, respectively (p=0.64). Significant differences in baseline characteristics included: antimicrobial indications of intraabdominal, respiratory, sepsis and febrile neutropenia; having a broad differential; and an intervention category of de-escalation.

**Conclusion:**

Direct communication with clinicians (eg. telephonically) improves ASP intervention acceptance rates compared to passive communication styles (eg. text-page). Direct communication with the prescriber encourages shared decision making, increases visibility of ASP, and allows for education to modify prescribing behavior. The success of an ASP relies heavily on provider relationships, which direct communication can further promote.

**Disclosures:**

**All Authors**: No reported disclosures

